# RESPOnSE—A Framework for Enforcing Risk-Aware Security Policies in Constrained Dynamic Environments

**DOI:** 10.3390/s20102960

**Published:** 2020-05-23

**Authors:** Christina Michailidou, Vasileios Gkioulos, Andrii Shalaginov, Athanasios Rizos, Andrea Saracino

**Affiliations:** 1 Istituto di Informatica e Telematica, Consiglio Nazionale delle Ricerche, 56124 Pisa, Italy; andrea.saracino@iit.cnr.it; 2 Department of Information Engineering, University of Pisa, 56122 Pisa, Italy; 3 Department of Information Security and Communication Technology, Norwegian University of Science and Technology, 2815 Gjøvik, Norway; andrii.shalaginov@ntnu.no; 4 Department of Computer Science, University of Pisa, 56127 Pisa, Italy; athanasios.rizos@di.unipi.it

**Keywords:** constrained dynamic systems, multicriteria decision making, policy based management, security, topsis

## Abstract

The enforcement of fine-grained access control policies in constrained dynamic networks can become a challenging task. The inherit constraints present in those networks, which result from the limitations of the edge devices in terms of power, computational capacity and storage, require an effective and efficient access control mechanism to be in place to provide suitable monitoring and control of actions and regulate the access over the resources. In this article, we present RESPOnSE, a framework for the specification and enforcement of security policies within such environments, where the computational burden is transferred to high-tier nodes, while low-tier nodes apply risk-aware policy enforcement. RESPOnSE builds on a combination of two widely used access control models, Attribute-Based Access Control and Role-Based Access Control, exploiting the benefits each one provides. Moreover, the proposed mechanism is founded on a compensatory multicriteria decision-making algorithm, based on the calculation of the Euclidean distance between the run-time values of the attributes present in the security policy and their ideal values, as those are specified within the established policy rules.

## 1. Introduction

Constrained dynamic networks [1] consist of small IT devices, named constrained nodes [2], which have limited resources for memory, computational capability and power. Internet of Things (IoT), emergency response, military operations and remote ecosystem monitoring are common areas where such devices and networks are frequently utilized, seeking to provide connectivity and access to services. In such networks, several sensors, actuators, devices, applications and end-users are interconnected, continuously producing and sharing data, information and resources. The nature of those data might be characterized as sensitive since they include and are not limited to personal information of the users, classified state and military operational documents, financial documents, etc. In addition, a recent report by F-Secure [3], regarding attacks over IoT, shows that only in the first six months of 2019, they measured over 2.9 billion events. Therefore, it becomes evident that constraint environments remain a relevant application domain which still needs the attention and the effort of the research community, since they are still prone to attacks and the safety of the assets may be jeopardized. To this end, in order to protect those data and to ensure that only authorized entities will have the right of access, a set of policies needs to be established and an efficient mechanism needs to be in place to enforce them. For example, if an organization wants to restrict and regulate access over a set of documents related to their finance department, then the security administrator can define policies which utilize data from different resources. He/she can control the total status of the network, denying the access if there is a threat present, the role of the subject who requests the access, the status of the document, the area from which the request is made, etc.

However, those environments present a high dynamicity, which results from the fact that new devices are continuously connected and disconnected from the network and new services are deployed, or existing ones are modified. These constant and unpredictable changes of the environment alongside the amount of information being produced, stored and transferred across such networks, increases both the complexity and size of the deployed security policies [4]. Moreover, the fact that the edge nodes generally present substantial constraints, in terms related to the size of memory and buffers, computational power, battery life and available interfaces [2], can also be an obstruction in the policy enforcement. Thus, protecting and regulating access over the data, as mentioned above, becomes a challenging task [5,6,7,8]. Managing security policies in such an environment requires an effective and efficient access control mechanism to be in place in order to provide suitable monitoring, control and audit solutions for managing data flows across communication and control links but also access to services.

Moreover, considering the complexity of constrained nodes and dynamic network applications, the chosen access control mechanism should be able to support complex policies which take into account attributes belonging to several domains. To this end, Attribute-Based Access Control (ABAC) [9] could be used in order to express security policies in such kinds of environments and regulate the access over a set of assets, by considering and evaluating multiple attributes related to the subject, the resource and the environment. However, the dynamicity of the aforementioned environments requires the definition of a large number of policies to ensure that all possible events will be taken into account in order to grant or deny access to a specific resource. However, the evaluation of the plethora of attributes usually present in ABAC policies demands the availability of operational resources which, as mentioned above, are limited. Hence, to reduce complexity, it is possible to exploit the Role-Based Access Control (RBAC) [10]. RBAC simplifies the process of writing security policies and reduces the complexity since the only considered attribute is the subject role. Although, in highly dynamic systems RBAC does not provide the necessary scalability and it causes an intrinsic reduction of expressiveness since several attributes and factors that can affect the access context and lead to misuse of the resources are not considered, and thus several conditions cannot be expressed. Moreover, RBAC systems can turn to be insufficient in capturing real-time changes which can potentially affect the access context, such as cyber-attacks over the network or the risk level of the subject requesting access. Thus, being able to enforce conditions with the same expressiveness of ABAC while at the same time keeping the simplicity and the low complexity which an RBAC model provides can result in an efficient and scalable access control mechanism, suitable for enforcing security policies in the environments above.

In this article we present RESPOnSE: a framework for the specification and enforcement of security policies within constrained dynamic networks based on a combination of both ABAC and RBAC, which exploits the benefits offered by both of them, to provide an efficient and at the same time scalable policy enforcement mechanism. RESPOnSE has been developed to provide effective fine-grained security policy-based management, still respecting the operational constraints of constrained dynamic networks. The operational workflow of RESPOnSE framework consists of two distinct phases, which are presented in detail. At first, the initialization phase in preparation for policy deployment take place, followed by a comprehensive description of the processes involved in the run-time operation. During the initialization phase, the assets under protection are classified into a number of predefined classes based on their specific object properties. For each one of the derived groups, a set of roles is defined as well as the necessary security policies per role. Those policies define the permissions and the access privileges of the specific role over a particular group of assets. Afterwards, in the run-time phase, RESPOnSE considers the attributes related to the subject and the environment in order to extract the appropriate role for this subject. In order to achieve this, a compensatory multicriteria decision-making algorithm has been utilized which is influenced by the Technique for Order of Preference by Similarity to Ideal Solution (TOPSIS). The outcome of the algorithm represents the optimal compromise solution, which is based on the calculation of the Euclidean distance between the run-time values of the subject and the ideal values, as those are specified within the established policy rules. Hence, the proposed solution exploits the advantages of ABAC by considering and evaluating attributes related to the subject and the access context in order to extract a permissible role for the subject. Having this role, it enforces RBAC policies, respecting thus the limitations of the constraint dynamic networks.

The contributions of this work are:

- We present RESPOnSE, an efficient and scalable policy enforcement framework, which is designed for environments with inherent constraints, such as IoT which includes edge nodes with limited resources. The proposed framework presents an innovative way of performing an access control procedure by dividing the operational workflow into two distinct phases. The initialization phase which can run in nodes with more computational power and the run-time enforcement of the policies, which can be handled by the constrained nodes of the network, providing thus a way of overcoming existing limitations and implementing fine-grained policy enforcement.
- The framework combines the ABAC and the RBAC models, utilizing the expressiveness of ABAC, during the initialization phase, by considering and evaluating the full set of subject and environmental attributes in order to extract and assign a role to the subject performing an access request. Following, during the run-time phase we use the extracted role in order to enforce RBAC policies for making the final access decision.
- For the extraction of the role the framework utilizes a modified version of the well known Multi-Criteria Decision Making (MCDM) algorithm, TOPSIS. In general, these kinds of algorithms rely on a Decision Matrix, which is constructed by a decision maker who gives a level of preference to each alternative with regard to each defined criterion. Instead, in this modified version, the Decision Matrix consists of the exact values of the attributes as they are defined in the security policy. Thus, the Decision Matrix can be generated automatically from the policy and the defined permissible roles and does not include the subjectiveness, which could be present if a decision-maker was setting the values. Moreover, a Current Value matrix is introduced, including the values of the attributes at the moment of the request or any upcoming re-evaluation. Hence, the best alternative, which in the proposed framework represents the role to be given to the subject, is considered the one which has the minimum geometric distance from the aforementioned matrix.

The rest of the article is structured as follows. Section 2 presents related work in the corresponding areas, Section 3 describes in detail the proposed model and the underlined assumptions and requirements, also analyzing all the necessary steps and processes per phase. Section 4 includes a use case example and, finally, Section 5 concludes the study.

## 2. Related Work

A number of techniques have been developed over the years, aiming at overcoming the limitation of human processing capabilities and providing to the decision-makers the possibility of structuring complex problems and evaluating all the available alternatives. These methods belong to a category of algorithms known as Multi-Criteria Decision Analysis (MCDA) and are briefly presented below. A comprehensive survey on the MCDA and their various categories was presented by Greene et al. in [11]. Two main categories which can be identified are the Multi-Attribute Decision-Making Methods (MADM) [12] and the Multi-Objective Decision-Making Methods (MODM) [13], where the first has a single objective evaluate a number of different criteria and attributes and the second is used in problems with multiple, often conflicting, objectives.

The application area of MCDM methods is wide and has been explored in detail in the study of Aruldoss et al. [14]. A few of the environments where such techniques were used include (i) the organization and management of a business, by tackling issues such as the selection of the proper business location [15] or the best supplier [16], (ii) in the banking system, where MCDM methods are exploited from evaluating the bank performance [17] up to the assignment of a credit limit per customer [18] and, finally, (iii) for performance evaluation within an organization either of the employees or of the provided services and the final profit [19]. Utilization of such methodologies can also be found in the information security domain, providing solutions mainly related to the risk assessment procedure of the organizations’ assets [20,21,22,23,24], while no work relevant to security policy-based management has been identified in the literature. A relevant work was also presented earlier by the authors in [25]. However, that study makes use of AHP and does not consider the additional limitations arising in highly dynamic and constrained environments. Respecting the aforementioned studies, in this paper the proposed model exploits a TOPSIS-based algorithm whose outcome forms the input of a risk-aware security policy enforcement procedure.

Moreover, several studies in the area of IoT make use of MCDM algorithms [26,27,28] either for efficiently managing the spectrum of IoT devices or assessing the influential factors in IoT-related enterprises. In addition, in the area of constraints environments such as IoT, there are studies trying to tackle the challenge of policy enforcement. In [29], the authors propose an access control framework for a body sensor network, while in [30] the authors present a standardized network security policy enforcement architecture for IoT devices. Respecting the aforementioned studies, in this paper, the proposed model exploits a TOPSIS-based algorithm whose outcome forms the input of a risk-aware security policy enforcement procedure.

Nevertheless, in real-world problems, which often contain erroneous, mistaken and noisy data, there is a strong need for advanced data analysis methods capable of handling such data irregularities and providing adequate support to MCDA methods in making decisions regarding security-related controls. Especially, this is important in security policy-based management based on the input data and attributes from distributed dynamic systems and networks that require precise decisions with low latency and sufficient flexibility in response to changes in the environment. The increased complexity of systems will eventually make human experts less efficient in decision making and therefore demand new approaches such as the Hybrid Intelligence (HI) methods [31,32]. The main motivation of such methods is to find a right trade-off between transparency, accuracy, resources complexity and interpretability of the extracted policies for later decision support [33]. As one of the most commonly used approaches, Fuzzy Logic (FL) offers transparency and interpretability of the model, while it is highly susceptible to input data errors and the complexity of the data. Such obstacles can be mitigated while giving a synergy with Neural Networks, which do not require prior information about the problem and can result in a high-level abstraction model with lower complexity in comparison to other Computational Intelligence (CI) methods. Therefore, two stages Neuro-Fuzzy (NF) HI method [34] can be seen in the information security applications as one of the most commonly used methods. NF can also be considered as a representative of so-called Soft Computing techniques, which can tolerate imprecision and inconsistencies among the real-world data. It is capable of finding automated data analytics and patterns, extracting human-understandable rules and assisting in decision support by providing both accurate classification and explanation of the decision process.

The RBAC and ABAC combination is a topic explored in several studies in the existing literature. For example, in [35] the authors present an access control model for web applications where the main access control model is RBAC but at the same time ABAC is also used in order to enforce attribute-based policies and control the set of permissions. Another attempt of combining the two models is the one of [36] where the authors proposed RABAC, a role-centric attribute-based access control model. The main idea of RABAC is the separation of the access decision into two parts, where in the first RBAC is exploited in order to define all the permissions each user can acquire and then a filtering policy takes place in order to determine the actual permissions per user. A solution of how attributes can be incorporated in an RBAC model is also provided in [37], where the authors propose three different ways to achieve it, while in [38] a formal definition of the RABAC model is provided as well as an extended study on the evaluation indicators and the efficiency of the model. The aforementioned studies provide solutions which are based on the integration of the two access control models into one unified model. Although, a hybrid model such as the aforementioned will not be suitable for the constrained application environments that we consider in our study. Thus, in our approach the two models are working in parallel with the higher-tier nodes utilizing the expresiveness of ABAC by considering a full set of attributes in order to extract a role, which is then being evaluated for the final decision. This choice was made because on one hand ABAC having to evaluate a bigger number of attributes requires more time [39] and consequently electrical and processing power, which is available in higher-tier nodes, and on the other hand lower-tier node can still exploit RBAC, which is a model frequently utilized in those environments [40,41,42,43] for the final decision.

## 3. Risk-Aware Security Policies Enforcement Mechanism (RESPOnSE)

This section presents RESPOnSE, a mechanism for the enforcement of risk-aware security policies within constrained dynamic systems, such as those described earlier. The operational workflow of the proposed solution is divided into two phases, the initialization and the run-time. The initialization phase is an essential element of RESPOnSE since it guides through the given procedure the security administrations towards the predeployment phase, which provides the prerequisites to run-time phase, where the enforcement of the security policies comes to be. It is worth noting that the proposed solution does not consider mainly Machine 2 Machine devices; instead, RESPOnSE considers systems that actively (e.g., changing physical values in the user environment) or passively (e.g., collecting user’s data) interact with the human user. Moreover, RESPOnSE handles data access in a multitenant environment, i.e., an environment where devices from more than one provider are present, hence it is not possible to perform strong trust assumptions.

The mechanism is utilized at the edge nodes deployed within the proximity network of such systems, while the computational burden is transferred to the policy specification and predeployment phases for the initialization and to higher tier nodes during run-time. The described operations have been developed in accordance with the following requirements:

- Policy rules capture multiple attributes that are mutable in run-time and can be related to the subject, the object or the environment. Examples of those attributes could be the time, the location, the status of the network, the risk level of the subject, etc.
- Each attribute is associated with a criticality metric. Thus, the impact of an attribute in the final decision can be more or less significantly lower depending on the resource that the security policy protects.
- Each attribute is associated with a freshness metric. Since, as mentioned above, the policy rules may capture mutable attributes, a freshness metric is associated with each one of them in order to designate how often a retrieval of the attribute values will take place.
- The possible types and ranges of the attributes are only limited by the specification language.
- High-tier nodes have the required resources for the enforcement of fine-grained security policy management, in accordance with the traditional Conditions → Rules → Capabilities paradigm.
- Low-tier nodes, which lack in computational power, size of memory, battery life, such as for example IoT devices, enforce risk-aware security policies, based on the following assumptions:
  - Assets can be classified into a predefined number of classes in accordance with a delimited number of axioms and their specific data/object-property values. Assets can be identified as the resources that the security policy is meant to protect, as for example a set of classified documents, military operational documents, personal information of the users, etc. The axioms are defined in order to establish the attributes of those assets, allowing their classification in accordance with the similarities of the preconditions governing their specific action requirements.
  - Each subject can be assigned a dynamic role for each asset class in accordance with the real-time values of its relevant attributes. The role is inferred at high-tier nodes (who in run-time enforce Dynamic ABAC per asset), and it is delivered to low-tier nodes (who in run-rime enforce risk-aware Dynamic RBAC per asset class).
  - The utilized classification mechanism must provide membership functions for the classification of new assets, or reclassification of assets with mutable attributes, without requiring continuous retraining for minor changes in addition to the periodic maintenance.
  - The utilized classification mechanism must allow the forced assignment of a specific asset within a predetermined class (even a singular class) based on a criticality metric, in order to support tailored security policy-based management for specific assets.
  - The assigned subject role must be able to be reinferred in run-time, both as a periodic process and as an event-triggered process.
  - The security administrators must be allowed to precisely define the permissible margins for subject role assignment.

Accordingly, the risk is integrated by the security administrators in order to support fine-grained security policy-based management at the constrained edge nodes deployed at the proximity networks of constrained distributed dynamic systems, such as those described earlier. Risk is instantiated in the following processes:

- Grouping assets into classes.
- Assigning roles to subjects in accordance with the run-time values of mutable attributes, allowing for constrained elasticity to the exact permissible values.
- Defining security policies in a per-class/per-role basis.

The following subsections present the developed mechanism for the satisfaction of these requirements. As mentioned, the operational workflow is divided into two distinct phases, which are thoroughly described.

### 3.1. Initialization Phase

**Figure 1:**
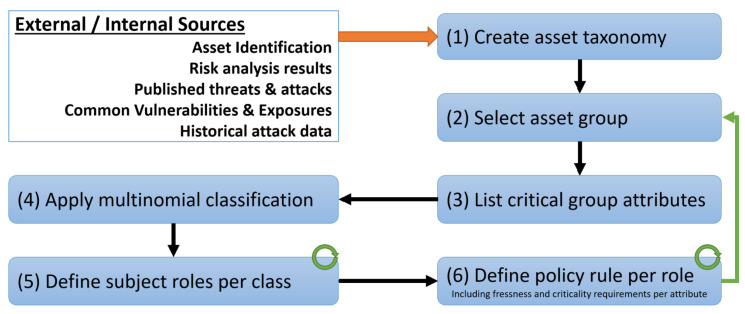
Process diagram for the initialization phase.

Figure 1 depicts the process that the proposed mechanism uses during the initialization phase, in order to classify the assets, establish permissible subject roles and define the necessary policies per role so as to regulate access. In the defined process, steps 2, 5 and 6 are cyclical in order to ensure policy completeness, as described below in detail.

**Mechanism—step 1: Create Asset Taxonomy:** The initial step refers to creating an asset taxonomy, at a level of detail that is sufficient to support the definition of fine-grained security policies. Inputs for this step can arise from formal asset identification processes [44], earlier risk analysis results, published common vulnerabilities and exposures or enterprise internal historical attack data. Depending on the required granularity level, such a taxonomy can be formalized using knowledge representation methods such as ontologies or conceptual graphs.

**Figure 2:**
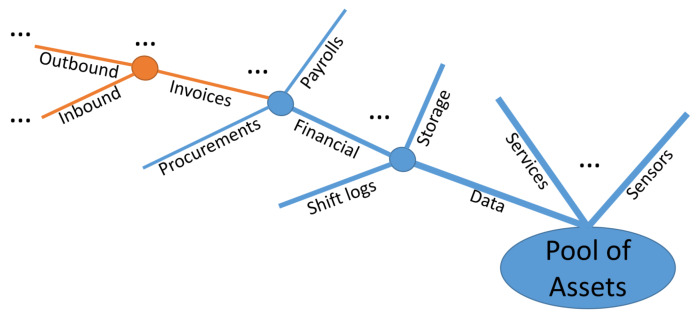
Selected branch of asset taxonomy.

**Mechanism—-step 2: Select Asset Group:** Step 2 is the first cyclical process, where branches and individuals belonging to specific classes require policy separation from their parent nodes. This separation relies on the initial inputs from external/internal sources but also on operational conditions affecting the required policy granularity. Figure 2 depicts a possible asset taxonomy that can be created within an organization, including but not limited to data, services, sensors, etc. Following the branch related to the data, the separation of policies can be enforced at the level of (i) Inbound/Outbound Invoices; (ii) Financial documents; or (iii) Overall Data, defining and applying the same access control policies for the individuals belonging to the selected class and all their children nodes. The process is cyclical so that all the children nodes have been selected and suitable policies have been specified, ensuring completeness in this way.

**Mechanism—step 3: List Critical Group Attributes:** In step 3, the security administrator creates each asset as an individual within the defined classes and lists its critical attributes by defining axioms that establish its type, relationships and object/data properties. This is established within the aforementioned taxonomy but can be graphically represented (or also constructed depending on the system complexity) as presented in Table 1, where F($X_{k}$,$Y_{n}$) indicates the correlation among the asset and its attributes and the example presented in Table 2. For the purpose of this example, the chosen asset group is the invoices which belong to the financial data of a company. Each of the assets has a unique identifier and is associated with a number of attributes. Furthermore, each attribute can acquire a value within a specified range and is associated with a certain level of criticality. For example, we can assign a high level of criticality to the classification attribute and a low-level criticality to the region attribute.

**Figure 3:**
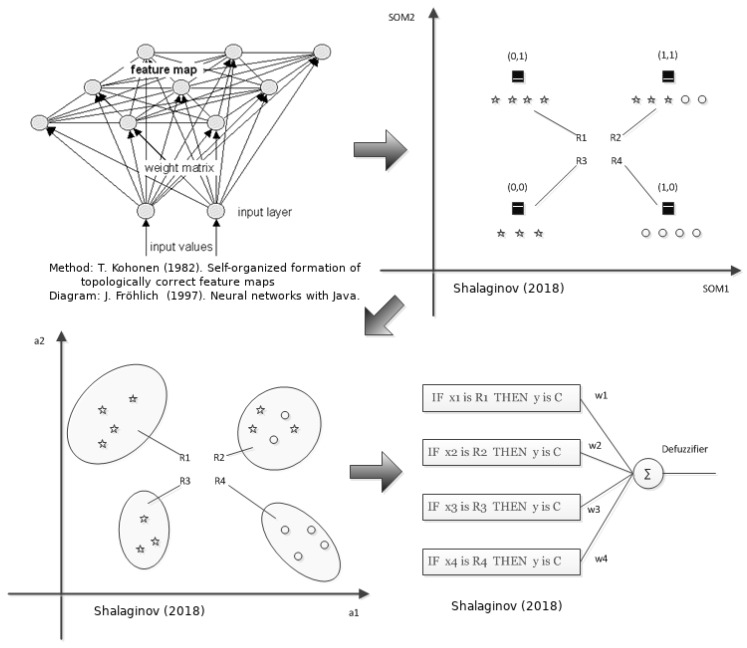
Neuro-Fuzzy representation.

**Mechanism—step 4: Apply Multinomial Assets Classification:** Multinomial classification is applied to the assets belonging to the defined groups, in order to identify classes based on the similarity of their attributes and criticality values. NF is a two stages HI method that assembles FL and Artificial Neural Networks (ANN) into an intelligent model, shown in Figure 3. The major data operations of NF are divided into two logical stages [33], as shown in Figure 3.

**1st NF stage** is an unsupervised procedure that is aimed at grouping samples according to their similarity using Self-Organizing Map (SOM) or so-called Kohonen map. SOM is one of the most powerful Neural Network-based unsupervised models. Among various applications, it is used for 2D data representations and data grouping of multidimensional input data. The main difference from clustering is that the input data samples are grouped according to their similarity not just by measuring the distance between a cluster center and any of the samples. This model was originally proposed by Teuvo Kohonen [45], where he described self-organizing systems as a one- or two-dimensional matrices that have a feedback connection between neighboring nodes in the map. The resulting formations of such maps depend on the feedback level and order of the samples being fed into the map-making, possibly automated formation of the similarities from map topology. This step includes the four following substeps:

1. *Clustering based on the features similarities*. The input data sample is a real-valued vector $X=[x_{i}\in R,\leq i\leq M-1]$ with a corresponding set of features $X=\{x_{0},\cdots ,x_{M-1}\}$ and characterizes a point in *M*-dimensional features space. Once SOM is trained, this will result in a set of groups containing very similar samples.
2. *Construction of elliptic regions*. To be able to characterize extracted data groups in a common manner, the hypothesis is being set that the following multivariate distribution defines the model of the data in a particular group [46] extracted from the SOM node $S_{i,j}$:
  (1) $$g\left(x\right)=\frac{1}{\sqrt{{\left(2\cdot \pi \right)}^{M}\left|\Sigma \right|}}\cdot e^{-\frac{1}{2}{\left(x-\bar{x}\right)}^{T}\Sigma^{-1}\left(x-\bar{x}\right)}$$
  In this step, we put an assumption based on the data sample set that the features distribution is a Gaussian/Norwam one. To test this, a generalized Pearson $\chi^{2}$ test for multidimensional data is being used. The $\chi^{2}$ value will reflect the probabilistic radius of the hyperellipsoid (Mahalanobis distance) from the centroid of cluster to any point in the distribution:
  (2) $$\chi^{2}=\sum_{n=0}^{M-1}\frac{{\left(x_{i}\left(a_{n}\right)-E\left(a_{n}\right)\right)}^{2}}{E(a_{n})}$$
  where the $E(a_{n})$ represents a theoretical expectation of the particular feature $a_{i}$ and equals to sample’s mean $c_{i}$. By means of ranging of the continuous variables, the $\chi^{2}$ statistics can be calculated based on degrees of freedom, taking into consideration number of features *M*.
  This statistical model defines “goodness of fit” [47] of the data samples in the hyper elliptical region by means of $\chi^{2}$ distribution test. It describes how well the distributed data samples fit the defined multivariate distribution. $\chi^{2}$ distribution roughly is a sum of the squared difference between all points in a given set. To determine the value of the $\chi^{2}$ based on the β and degrees of freedom, a contingency table is being used. Furthermore, the squared radius of the hyperellipsoid is equal to the $\chi^{2}$ considering the equation above.
3. *Extracting the parameters of the fuzzy patches/fuzzy logic rules*. A first stage of the Principle Component Analysis (PCA) is being utilized to extract the set of eigenvalues $\bar{\lambda }$ and set of eigenvectors $\bar{\bar{v}}$ [48]. With the help of PCA we rotate the original multidimensional distribution to remove the correlation. This is done since the distribution might have unequal deviations and directions different from the main feature axes. The aforementioned set of parameters has to be defined as the complete characteristics of the fuzzy patch. Therefore, we can specify each feature region as $\Pi^{i}$ that defines the fuzzy patch. This is done since at the current step, it is hard to understand the heuristics behind this region formation, yet possible to understand similarities by such a fuzzy patch definition.
4. *Construction of membership function*. Each fuzzy patch is characterized by the membership function or so-called “degree of truth” that binds an input with an output [49]:
  (3) $$\mu_{R}=e^{-\frac{1}{2}{\left(x-c\right)}^{T}\;P\;\Lambda \;P^{T}\;\left(x-c\right)}$$

**2nd NF stage** is a supervised procedure that is used to fine-tune the final classification model based on the descriptive fuzzy logic rules extracted in a previous step. On this stage, a set of fuzzy rules (based on fuzzy patches) are fed to ANN with their corresponding weights assigned. The iterative training procedure results in an accurate classification model employing the aforementioned fuzzy rules. Once all the fuzzy patches discovered (also called groups of data samples), the descriptive part is fixed. Further on, the model fitting is performed by the Delta Learning rule in order to reduce errors between the labeled data samples and those predicted by the descriptive part.

It is worth noting that the general clustering is a family of unsupervised learning methods, which are divided into two subcategories (i) partitioning or (ii) hierarchical. In either of those, it is essential to know the initial parameters: exact number of final clusters in addition to most likely centroids placement in (i). This will make the unsupervised approach very much dependent on the knowledge of domain expert who should select and evaluate such parameters. However, this can be a challenging task due to time limitations and the existence of the Big Data paradigm, especially in cybersecurity tasks in dynamic environments. The suggested methodology is based on the so-called similarity grouping, giving the property of placing the data samples in the same neighborhood through Best Matching Units (BMU) in Self-Organizing Map as 1st NF stage. The 2nd NF stage offers so-called fuzzy matching, where an object can be part of several groups with different level of “degree of truth”, which makes it more practical in real-world data where such an approach is preferable to the “crisp” approach (like binary logic), including IoT devices scenario and data processing.

**Mechanism—step 5: Define permissible subject roles per class:** Step 5 is devoted to defining the roles for each of the established classes. These roles will define the privileges that the subject has and the actions that he/she can perform within a specific class. The number of roles that can be assigned to each class is decided by the security administrator and depends on the (i) constraints arising by the application environment, the (ii) operational requirements of the system and the (iii) criticality of the assets that have been assigned to the specific class. It is worth mentioning that the same role can be assigned to more than one class, while the security policies governing such roles can be identical or discrete. For example, supposing that after following the procedure in step 4 the NF method produces three distinct classes, the subject roles can be defined Class A → Guest, Employee, Manager, Class B → Customer, Intern, Class C → Guest, Operator

**Mechanism—step 6: Define policy rule per role:** In step 6, security policy rules are defined in a per-role/per-class basis. Those rules are utilized during run-time for the assignment of roles to each subject requesting access to any of the assets belonging to a specific class. Rules are constructed based on the subject and environmental attributes, the values of which are evaluated and a role is assigned to the subject for all the assets of a specific class. For example, the security administrator can define a policy rule for Class A of the previous example with the following form: *“For Class A, if the level of expertise of the subject is expert, the department is Finance, the working region is not Europe and the subject does not have a clearance penalty then the role that will be assigned to the subject is Manager”*. This can be formalized using description logic as:

Manager≡hasExpertise.Expert⊓hasDepartment.Finance⊓hasWorkingRegion.¬Europe⊓hasClearancePenalty.⊥

It is worth mentioning that, as reported in [50], the formation and the definition of a security policy always involves a number of people. Those are usually people responsible for the security of the system, technical stuff, people from the legal department, etc. The choice of involving all those stakeholders in the procedure of defining the security policies ensures the robustness of the policies and the fact that all aspects have been taken into account for the protection of the assets. Therefore, in a policy specification framework the human involvement is necessary from the risk assessment and analysis up to the final definition of the security policies and the policy translation. However, many times the interaction of different stakeholders may lead to mistakes or misconfiguration. Hence, although outside the scope of this article, it must be stated that syntactic, terminological and conceptual homogeneity must be maintained between the risk-aware policy rules enforced in low-tier nodes and the fine-grained policy rules enforced in high-tier nodes of the same system. Accordingly, suitable policy aggregation and policy deconflictation/reconciliation mechanisms must be employed during this step to support the policy administrators. To this end, a number of policy verification and validation frameworks have been proposed in the literature [51,52,53,54] and can be exploited in order to eliminate any mistakes and misconfigurations. As a final point, it is worth mentioning that the policy specification cannot be considered as a static procedure. The needs of the application environment might change over time or an incident may happen and the policies must be adapted accordingly in order to continue providing the necessary protection of the assets. Thus, as stated in [50], a review of the policies and the procedures on an regular basis is necessary. Although, the reconfiguration of the policies is not a procedure that will take place often and thus the proposed configuration phase of RESPOnSE will not burden the system.

### 3.2. Run-Time Phase

The run-time phase can be divided into two steps, (i) the extraction of the role and (ii) the assignment of the access rights to a specific role based on the given policies.

**Mechanism—step 7: Role Extraction:** In this step, the subject who requested access to one of the assets belonging to one of the classes derived from step 4 of the proposed mechanism will be assigned a specific role for this class. As mentioned, RESPOnSE utilizes both ABAC and RBAC. ABAC is exploited in the higher-tier nodes in order to extract a single permissible role for the subject. This extraction is based on the run-time values of a set of attributes which are related both to the subject (i.e., the department to which the subject belongs, the type of equipment used, etc.) and the environment (i.e., the time of the request, the location, etc.). The possible values, types and ranges of the attributes are predefined and only limited by the specification language. It is worth mentioning that attributes can also be included as typical RBAC elements such as the hierarchy of the subject. Hence, we exploit this expressiveness of ABAC in order to extract the role which then will be pushed to lower-tier nodes. Thus, by having to evaluate just a single attribute we keep the complexity and the need for computational resources to a very low level.

Assigning one of the permissible roles of a class to a subject is handled as a Multi-Criteria Decision Making (MCDM) problem. In this kind of problem, having defined a specific goal, there is the possibility of choosing the best alternative that meets this goal amongst a set of alternatives, considering multiple distinct and often conflicting criteria. For the purposes of this study, we consider as goal the extraction of the role, as alternatives the permissible roles of each class and as criteria the attributes which are encapsulated to the specific policy rules established in step-6.

The proposed mechanism is a compensatory multicriteria decision analysis method, which has been influenced by the Technique for Order Preference by Similarity to Ideal Solution (TOPSIS) [55] method, which is widely used for modeling and solving MCDM problems, and it is also applied in a number of studies in the area of constraints environments and IoT [56,57,58]. The fundamental assumption of TOPSIS is that the most suitable alternative must have the shortest geometric distance from the positive ideal solution (PIS) and at the same time the longest geometric distance from the negative ideal solution (NIS). The PIS and the NIS are calculated considering the values of the best (benefit) and the worst (cost) attributes, respectively. In the proposed mechanism none of the attributes is characterized as costly; therefore, estimating a NIS is not relevant. Although a relative weight can be assigned to each attribute so as to define its importance for the final decision, none of them can be seen as cost-oriented. Hence, the best alternative is considered the one which has the minimum geometric distance from the run-time values of the under evaluation attributes. Therefore, as Ideal Solution, we consider a Current Value (CV) matrix including the values of the attributes at the moment of the request or any upcoming re-evaluation.The developed mechanism can be divided into a number of steps which are briefly described below.

**Figure 4:**
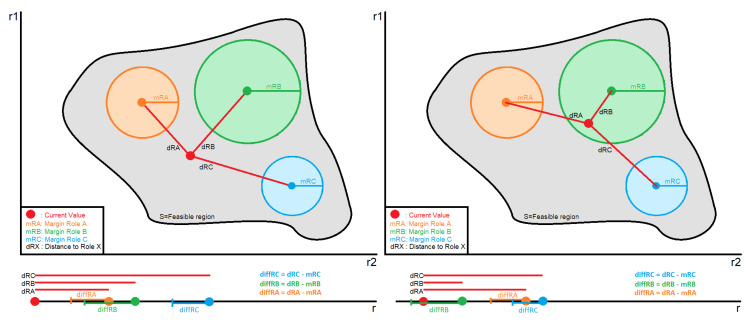
Role assignment to subject.

1. Construction of the Decision Matrix (DM)
  The construction of the DM can be expressed as shown in Table 3, where $A_{1}$, $A_{2}$,..,$A_{m}$ refer to the set of the alternatives of the given problem, $C_{1}$,$C_{2}$,...,Cn refer to the set of the criteria on which the final decision is based and finally $x_{ij}$ is the level of preference for the alternative $A_{i}$ with respect to the criterion $C_{j}$.
  In the proposed mechanism the DM is constructed, taking as inputs the required values of the subject and environmental attributes that are encapsulated into the policy rules specified for the roles of the examined class. Hence $A_{1}$, $A_{2}$, ...,$A_{m}$ represent the permissible roles for this class, $C_{1}$,$C_{2}$,...,Cn represent the attributes that need to be evaluated for the assignment of a role, and $x_{mn}$ element of the matrix represents the exact required value of the Cn subject/environmental attribute, in order to assign the specific role. All these components are specified by the security administrator during the initialization phase, following the process described earlier.
2. Normalization of DM
  Before initiating the comparison among the criteria, a two-step preprocessing of the data must take place. The first one is to convert the values expressed in non-numerical terms into numerical ones. This can be achieved by giving a specific range of values per attribute, based on the nature of the attribute and the needs of the application environment. The second step is to identify the appropriate normalization technique so as to establish a common scale for all the criteria and be able to compare them. In the literature, there are a number of normalization techniques which can be exploited in the TOPSIS method [59], with the most applicable being the Vector Normalization [60,61]. However, for the proposed mechanism the chosen normalization technique will also be used for normalizing the CV matrix, which contains the run-time values of the attributes. Considering that the values may not be in compliance with the values defined in the policy and can fall in any point of the permitted range (e.g., if the subject belongs to an existing department that is not anticipated within the established policy rules for the class under evaluation), the utilized normalization technique should take into account the whole range of potential values. Thus, the aforementioned technique is limited only in the values which are present at the DM; it does not serve the scope of this mechanism. Instead, the proposed methodology exploits the Linear Max-Min technique [62], were given a range of values per attribute $\left[r_{min},\ldots ,r_{max}\right]$, the normalized values of the DM are calculated by the Equation (4), which considers both the current value of the DM and the full range of values that the specific element can acquire.
  (4) $$n_{ij}=\frac{x_{ij}-r_{min}}{r_{max}-r_{min}}$$
3. Definition of the weights
  The relative importance of each criterion can be established using a set of weights whose sum equals to one. Such a set can be defined as $W=\{w_{1},w_{2},\ldots ,w_{n}\}$, where $w_{j}$∈R is the weight assigned to criterion $C_{j}$. Even though appointing the appropriate values to the weights will not be investigated in this study, in the current literature and mostly in the field of Artificial Intelligence, this problem has already been addressed and a number of solutions are available. For instance, a relevant method is the backpropagation [63] which, given an initial set of weights and the error at the output, updates each of the weights in the network so that they cause the actual output to be closer to the target output over multiple training iterations. This procedure continues until an optimal solution is achieved. Another widely used way of solving the aforementioned problem is the genetic algorithms [64] which have been proven to be very effective as global optimization processes and not just finding local minima/maxima solution.
4. Calculation of the weighted normalized DM (WDM)
  The WDM is derived by the multiplication of each element of each column of the normalized DM with the relative weights derived from the previous step.
  (5) $$z_{ij}=w_{j}n_{ij}$$
5. Identification and normalization of the CV
  Ideal Solution is considered a matrix which can be defined as CV = [$v_{1}$,$v_{2}$, ...,$v_{n}$] and contains the run-time values of the evaluated criteria (i.e., subject/ environmental attributes). Therefore, the element $v_{n}$ of the matrix represents the value of the Cn criterion at the time of the evaluation.
  As previously stated, in order to be able to compare these values with the one of the WDM, the same normalization technique of step 2 will be applied to the CV matrix. Hence, using the Equation (4) we calculate the normalized CV as $CV=[y_{1},y_{2},\ldots ,y_{n}]$.
6. Calculation of the weighted normalized CV (WCV)
  Since both the DM and the CV matrices represent the values of the attributes, they have to be expressed with common terms. Thus, the same set of weights will be applied also in the CV matrix resulting in a (WCV) matrix.
  (6) $$WCV=\left[k_{1},k_{2},\ldots ,k_{n}\right]=\left[y_{1}\times w_{1},y_{2}\times w_{2},\ldots ,y_{n}\times w_{n}\right]$$
7. Calculation of the distance of each alternative from the WCV
  The distance is calculated using the Euclidean distance.
  (7) $$S=\sqrt{\sum_{j=1}^{n}{\left(z_{ij}-k_{i}\right)}^{2}},i=1,\ldots ,m$$
  Accordingly, this step provides the divergence of the current subject/environmental attribute values from those required for the assignment of each of the permissible roles.
8. Ranking of the alternatives and final decision
  The outcome of the previous steps will be the ranking of the given alternatives based on their distance from the CV, where the optimal alternative is the one with the shortest distance. Nonetheless, assigning the role with the shortest distance may introduce unacceptable risks. Although this can be partially mitigated by increasing the quantization resolution (i.e., by increasing the number of permissible roles), this is not an efficient solution and in most cases not desirable or permitted. Accordingly, the system administrator should be able to define ranges in order to quantify the acceptable distance for assigning a role. The threshold of these ranges will be based on the criticality of the asset, meaning that for very critical assets the maximum acceptable distance should be very close to CV, even 0. For assets which are not so critical, the margins can be extended, giving thus a higher level of freedom and more chances for a role to be chosen. Moreover, the closeness amongst the ranges is also configurable, so as to give the possibility to the security administrator to define, in a fine-grained way, the accepted margins of uncertainty when transitioning between roles. For example, if a subject by acquiring Role A is entitled to a set of privileges which provide full autonomy over the asset, then segueing into this role must be challenging. This can be achieved both by limiting the acceptable distance range of this role and at the same time by drifting apart the roles.
  Nevertheless, the procedure of choosing the correct values of the threshold is a challenging task, and it is discussed a lot in the research community. Thus, in this study, we refer to some methods which can be exploited to tackle this problem. The first one is the REINFORCE algorithm presented by Williams [65] based on which the authors of [66] extracted a learning rule which combined with an appropriate policy is able to set the most suitable thresholds for an optimal decision making. The second methodology depends on the use of Bayesian optimization [66]. This method relies on the mean reward and the variance of it applying a threshold, and then maximizing these gives a guide for selecting the appropriate threshold values in the future.
  Figure 4 depicts the assignment of the role to a subject considering the given margins from the security administrator. Each role can be represented as a circle, where the radius is the defined margin for this role. Moreover, the calculated values of distance matrix *S* in step 7 are denoted as the distance of the center of each role to the CV. Hence, we can calculate the difference between the distance of the role and the corresponding margin. If none of these differences is less or equal to zero (left graph), that means that none of the roles presents an acceptable distance from the CV so as to be assigned to the subject. In this case, the role is characterized as undefined and a default action defined by the administrator is performed (i.e., “Deny by default”, “Permit by default”). On the other hand, if one or more of the roles present a negative or equal to zero difference (right graph), the CV lies within the given margins of the role, meaning that the role presents an acceptable distance. Therefore, this role is the one assigned to the subject. It is worth mentioning that in case of an overlap, meaning that the CV lies within two or more circles, the role to be assigned is the one whose value in the distance matrix *S* is the smallest.

The aforementioned procedure for the role extraction can take place not only upon a request but also periodically so as to assure that the given roles assigned to the subjects are still valid. The time interval in which a re-evaluation will be performed is specified in accordance to two main criteria, namely (i) the rate of change of the specific attributes integrated within the policy rules (affecting their required freshness), and (ii) the confidence in the role assignment (in respect to the distance to the IS). The python code which implements the proposed component for the extraction of subject roles during run-time operation can be found online: https://github.com/chrismichailidou/topsisResponse.

**Mechanism—step 8: Assignment of access rights:** The final step of the run-time phase is the assignment of the access rights. Having extracted the role, the dedicated components of the policy administration and enforcement subsystem (provisioned in a high-tier node higher in the hierarchy) will send the answer to a Policy Decision Point (provisioned in the low-tier nodes). This component is responsible for storing the access rights per role to a Look Up Table (LUT), match the result obtained by the role extraction phase with the entries of the LUT and assign the appropriate rights to the subject upon request. Additionally, given that any given role is assigned to the subject for all assets belonging to the same class, a consecutive access request for an asset of the same class will not invoke a communication between the high and low tier nodes. Such communication occurs only in cases where:

1. Low to High tier node communication: an access request is received from a subject with no active sessions (therefore no role assignment) for an asset class.
2. High to low tier node communication: the freshness values of the attributes based on which the role of any given subject has been inferred have expired and require re-evaluation.
3. Low to High tier node communication: Some policy violation event (trigger event by the security administrator) triggers a role re-evaluation for a subject’s role.

Preliminary studies and evaluation towards such an enforcement architecture have been presented in earlier work [67,68].

## 4. An Example Use Case

For the purposes of this example, we based our analysis in data coming from a real company. As described earlier, before initiating the comparison among the criteria, we need to convert the values which are expressed in non-numerical terms into numerical ones. This can be achieved by giving a specific range of values per attribute, based on the nature of the attribute. For example, in the given use case, the departments of the company are in total 20. Thus, each one of them is assigned with a numerical value within this range. Having this mapping, we then move on with normalization of this matrix using the appropriate technique, as described in Section 3.

**Step-1**: The information security administrator of the company, having conducted an asset identification process and also having analyzed previous incidents within the company, created the Asset Taxonomy. This taxonomy includes assets such as various Data (i.e., Financial Documents, Customer Information, Customer Reviews etc.), Services (i.e., Customer Relationship Manager, Remote Diagnostics and Maintenance, etc.) and Sensors within the company’s HQ (i.e., temperature sensor, CCTVs, smoke detector, etc.).

**Steps-2,3**: The selected asset group and the one used throughout the evaluation is the invoices belonging to the financial data of the company. The asset group contains a total number of 150 invoices. Each one of the invoices is correlated with 25 attributes. A fragment of the structure of the asset group can be found in Table 2.

**Step-4**: Having the assets and the values of their attributes, step 4 of the proposed mechanism initiates. In this step, the assets are classified based on their similarities in five distinct classes. The level of the similarity is defined in accordance with their corresponding attributes. Hence, if two assets have the same values for a number of attributes, they will be classified in the same group. The input data were formed using the structure presented in Table 1. In general this step demonstrates:

- preliminary data analytics through looking at the distribution and correlation
- results of SOM training listing numerical ID of data samples assigned to each SOM node
- regressional performance evaluation of the method and given dataset and finally
- classification confusion matrix, showing how well the model actually can predict the data class and its actual class

**Figure 5:**
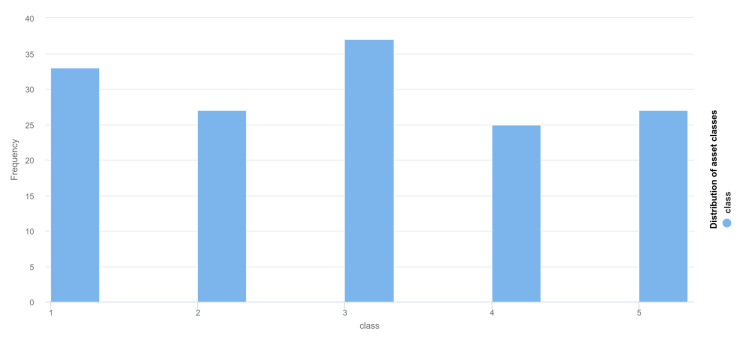
Distribution of asset classes.

**Figure 6:**
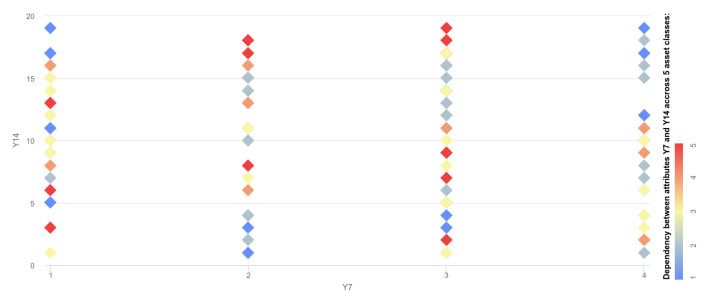
Correlation between attributes in dataset.

The technical specifications of the evaluation system are as follows: Intel Core i7-6600U CPU @ 2.60Ghz (2 cores: 4 threads and 5616 bogomips). 256GB SSD, 16GB DDR4 RAM. Neuro-Fuzzy method was implemented using C/C++ to achieve the best low-level memory performance using STL, Boost, Eigen, OpenMP libraries and multithreaded execution. Moreover, before performing experiments, the data have been analyzed with the community-accepted Machine Learning and Statistics software: Weka (https://www.cs.waikato.ac.nz/ml/weka/) and RapidMiner (https://rapidminer.com). The distribution of the classes is presented in Figure 5, while Figure 6 presents the distribution of the data across the various attributes. It can be seen that the data do not follow a specific correlation pattern, and it can be anticipated that the NF method will result in a few more general clusters.

*Parameters of the Neuro-Fuzzy method*. The method was parametrized as: number of features from the input dataset *M* = 25, confidence interval β=95% as most commonly used value, number of epochs of SOM training (1st NF stage)-150, number of ANN tuning epochs (2nd NF stage)-10. The last two parameters are selected based on the performance of the method and represent the general values used in community.

**1st stage NF results**. Based on the exploratory data analytic, the suggested SOM size was 4 × 2 nodes based on the method of optimal size determination for multinomial classification described in [33]. Following this, the corresponding unsupervised grouping of the input assets was performed for each of the SOM nodes:

- *SOMnode(0,0)*: 0 1 2 3 4 5 6 7 8 9 10 11 12 13 14 15 16 17 22 23 24 25 27 29 30 32 34 36 38 39 45 47 51 52 54 64 67 68 78 83 86 87 100 101 103 106 109 113 124 138 144 145
- *SOMnode(0,1)*: 20 42 48 50 57 60 61 62 71 72 73 75 88 89 94 95 99 114 115 127 131 136 139 146 147
- *SOMnode (1,0)*: 21 41 43 70 79 92 105 119 122 129 133 142
- *SOMnode(1,1)*: 18 93 118 120 128 132
- *SOMnode(2,0)*: 26 31 33 55 63 81 96 110 116
- *SOMnode(2,1)*: 19 28 35 37 40 44 49 58 59 98 108 111 121 134
- *SOMnode(3,0)*: 66 69 74 76 82 97 104 123 125 141 148
- *SOMnode(3,1)*: 46 53 56 65 77 80 84 85 90 91 102 107 112 117 126 130 135 137 140 143

Since the problem is a classification one, 30 general clusters have been extracted from the aforementioned assignment of SOM nodes.

**2nd stage NF results**. The extracted fuzzy rules with the help of the new method with Gaussian membership function described in [33] can classify 95.3020% of the assets properly, having Mean Absolute Percentage Error (MAPE) 2.5608% and Mean Absolute Error (MAE) 0.0555. At the same time, the standard triangular membership function method gave only 17.4497% classification accuracy with MAPE 57.1259% and MAE 1.1851, which is too low and cannot be used for a reliable result. Furthermore, the Confusion Matrix is given in Table 4. At the same time, the performance time complexity of the method is $8.69\cdot 10^{-5}$ s per asset, meaning the time it takes to find a corresponding fuzzy rule that can classify a new asset. Accordingly, this component of the proposed mechanism offers satisfactory performance not only for the initial asset classification (both at the initialization and run-time phases) but also for the reclassification of assets whose governing security policies integrate mutable attributes.

**Step-5**: The security administrator considering the several operations which take place within the company defines three roles per class and assigns to each one of them specific privileges. The roles and the corresponding privileges are given in Table 5.

**Step-6**: Finally, the security administrator defined the necessary policy rules in order to assign a role to each subject requesting access to any of the assets. These policy rules are based on the possible attributes that the subject can have, as well as on a number of environmental attributes. The evaluation of the policy rules results in the assignment of a role to a subject for the class he/she requested access to. For this test case, for Class A an example of the policies that have been defined are:

1. *Rule 1*: If the subject belongs to the Accounting and Finance Department, his identifier is within the 4893XXXX range, the time of the request is between 6 and 9 am and the connection type is through an Ethernet cable then the role of the subject is *Manager*.
2. *Rule 2*: If the subject belongs to the Marketing Department, his identifier is within the 8849XXXX range, the time of the request is between 5 and 7 pm and the connection type is through an Ethernet cable then the role of the subject is *Employee*.
3. *Rule 3*: If the subject belongs to the Production Department, his identifier is within the 5634XXXX range, the time of the request is between 12 and 2 am and the connection type is wireless with WEP encryption then the role of the subject is *Intern*.

For the rest of the classes, similar sets of rules were defined.

**Step-7,8**: A prerequisite which needs to be addressed before following the required procedure of the role extraction, is to assign to the attributes participating in the policy a numerical value within the given scale per attribute, so as to be in line with the demands of the exploited algorithm. For the purposes of this example, the considered ranges and corresponding values for each one of the policy attributes are as shown in Table 6.

Having these values, the following step is to construct the decision matrix, normalize it and define the weights to be applied for each criterion. Table 7 presents the DM matrix related to the given example. As depicted, the sum of the weights equals to 1, and for this case study, we chose to give a higher level of importance to the first two attributes, the Department and the Identifier.

As described in the methodology in this study, we assume that the Current Values matrix results from the run-time values of the under evaluation attributes upon the time of the request or any upcoming reassessment. For the purposes of this use case we assume the following requests coming from two different subjects:

*Subject A:* belongs to the Marketing Department, his/her Identifier is 48934583, the time of the request is 10am and he/she is connected to the network via an Ethernet cable.

*Subject B:* belongs to the Accounting and Finance Department, his/her identifier is 56349812, the time of the request is 10am and he/she is connected to the network via Wi-Fi.

Hence, the Current Value Matrices corresponding to the two subjects are respectively $CV_{A}=\left[5,5,4,1\right]$ and $CV_{B}=\left[6,8,4,7\right]$. The same normalization technique and the same weights are applied also to these matrices as denoted in Equation (4). Having identified and constructed the required matrices, through Equation (7) we calculate the Euclidean Distance of each alternative from the Weighted Current Value Matrix. The outcome of this calculation is depicted by a matrix $S=[d_{1},d_{2},d_{3}]$ where $d_{x}$ element represents the distance of the *x* alternative from the CV. For the given example the distance matrices are $S_{A}=\left[0.02,0.07,0.11\right]$ and $S_{B}=\left[0.06,0.03,0.10\right]$.

The final step of the procedure is to actually assign the role to the subject based on the previous results. As previously mentioned in the methodology, the security administrator, based on a number of factors (i.e., the importance of the asset), is able to define how rigorous the acceptable distance will be in order for a role to be assigned. Let us consider two scenarios. In the first scenario, the subjects request access to a class containing very critical invoices, which depict the financial situation of the whole company. Therefore, the administrator defined very strict margins per role, ensuring that the deviation from the CV will be the smallest. On the other hand, in the second scenario, the subjects request access to a class containing activity-specific invoices, allowing thus relative flexibility in the definition of the margins. Table 8 presents the margins per role as determined by the security administrator, while Table 9 shows the difference among them and the distance of each role from the CV (given in $S_{A}$ and $S_{B}$ matrices).

**Figure 7:**
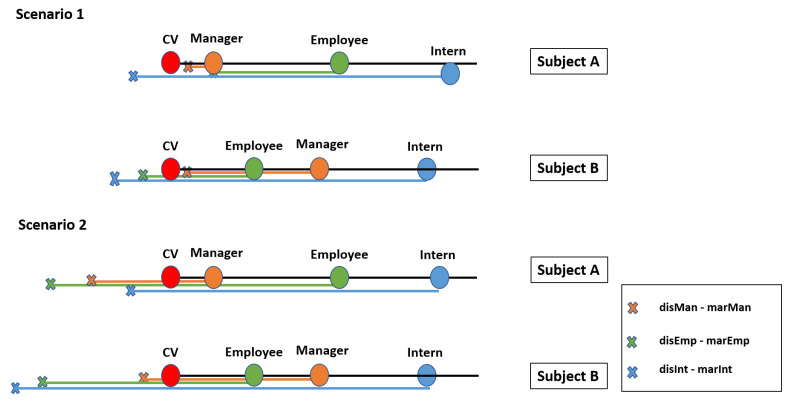
Role assignment.

At this point, the proposed algorithm will sort the distance matrices $S_{A}$ and $S_{B}$ in ascending order and will check sequentially if the difference of the calculated distance of the role from the CV and the defined margin for this role is less or equal to zero. The first role which satisfies this requirement will be assigned to the subject. The final results and the roles assigned to each subject per scenario are depicted in Figure 7.

Regarding the first scenario, for Subject A the role which presents a negative difference is the one of the Intern while for Subject B both the role of the Employee and of the Intern. In this case, the smallest distance belongs to the role of the Employee, and thus it will be evaluated first. Since the difference is smaller than zero, this is the role assigned to the subject. The same occurs for the second scenario for Subject A where one can observe that all roles present a negative difference. In this case, because in the distance matrix $S_{A}$ the role of the manager has the smallest distance, it will be evaluated first by the algorithm. Hence, the role of Manager will be assigned to the subject. Accordingly, for Subject B the first role to be evaluated based on the $S_{B}$ is the one of Employee, which presents a negative difference and thus will be assigned to the subject.

Consequently, as described in step 8, the policy administration and enforcement subsystem (provisioned in a high-tier node) transfer these roles to a Policy Decision Point (provisioned in the lower-tier nodes). This component is responsible for storing the access rights per role, matching the role to the predefined access rights and assigning them to the subject upon request.

## 5. Conclusions

This study presents RESPOnSE, a framework for the specification and the enforcement of risk-aware security policies in dynamic and constrained environments. RESPOnSE is based on a combination of ABAC and RBAC, taking advantage of the benefits that each one offers. The operational workflow of the framework is divided into two distinct phases, the initialization and the run-time. The first one includes the preparation for the policy deployment and processes such as the classification of the assets, the establishment of permissible subject roles and the definition of policies per role. In the second phase the proposed mechanism exploits a compensatory multicriteria decision making algorithm, influenced by TOPSIS, which by considering the actual values defined in the security policy, the run-time values of the subject at the time of the request or any upcoming re-evaluation and the criticality of the assets, computes the optimal compromise solution and assigns a role to a subject. Hence, the proposed solution exploits the advantages of ABAC by considering and evaluating attributes related to the subject and the access context in order to extract a permissible role for the subject. Having this role, it enforces RBAC policies, respecting thus the limitations of the constraint dynamic networks. As future work we intend to complete the implementation of a service architecture which is able to fully and independently support the proposed mechanism as presented earlier. Furthermore, we intend to develop a test-bed where it can be further evaluated in terms of performance under the constraints described earlier.

## Figures and Tables

**Table 1:** List of Critical Group Attributes.

Attributes → Asset Identifier ↓	Y1	Y2	...	Yn
X1	F(X1,Y1)	F(X1,Y2)	...	F(X1,Yn)
X2	F(X2,Y1)	F(X2,Y2)	...	F(X2,Yn)
X3	F(X3,Y1)	F(X3,Y2)	...	F(X3,Yn)
...	...	...	...	...
Xk	F(Xk,Y1)	F(Xk,Y2)	...	F(Xk,Yn)

**Table 2:** List of Critical Group Attributes—example.

Attributes → Asset Identifier ↓	Classification	Company	...	Department
Inv00013124	Secret	E.Factory	...	Financial.EF
Inv00015435	Confidential	Warehouse	...	Logistics.W
Inv00012343	Official	Logistics	...	Operations.L
...	...	...	...	...
Inv00019844	Restricted	R.M.Supplier	...	Productions.RMS

**Table 3:** Decision matrix.

	$C_{1}$	$C_{2}$	...	$C_{n}$
$A_{1}$	$x_{11}$	$x_{12}$	...	$x_{1n}$
$A_{2}$	$x_{21}$	$x_{22}$	...	$x_{2n}$
...	...	...	...	...
$A_{m}$	$x_{m1}$	$x_{m2}$	...	$x_{mn}$

**Table 4:** Confusion classification matrix: vertical—actual class, horizontal—predicted.

a/p	C1	C2	C3	C4	C5	Number of Assets
**C1**	33	0	0	0	0	33
**C2**	2	25	0	0	0	27
**C3**	2	0	35	0	0	37
**C4**	2	0	0	23	0	25
**C5**	1	0	0	0	26	27

**Table 5:** Roles and privileges per class.

Selected Class	Role Privileges Per Role		
Class A	Manager Read, Modify, Share	Employee Read	Intern No access
Class B	Manager Read, Modify, Share	Sys Admin Read, Modify, Share	Guest No access
Class C	Sys Admin Read, Modify, Share	Employee Read, Modify	Intern No access
Class D	CEO Read, Modify, Share	Employee Read, Modify	Guest Read
Class E	IT Read, Modify, Share	Stuff Read	Intern Read

**Table 6:** Assignment of numerical values to policy values.

	Range	Policy Value	Numerical Value
Department	1–20	Accounting	6
		Marketing	5
		Production	1
Identifier	1–100	4893XXXX	5
		8849XXXX	9
		5634XXXX	8
Time	1–8	6–9 a.m.	4
		12–2 p.m.	2
		5–7 p.m.	3
Connection Type	1–10	Ethernet	1
		WiFi	7
		4G	8

**Table 7:** Decision matrix and weights.

	Department	Identifier	Time	Connection Type
Manager	6	5	4	1
Employee	5	9	2	7
Intern	1	8	3	8
Weight	0.4	0.4	0.1	0.1

**Table 8:** Acceptable distance from Current Value (CV) per role.

	Manager	Employee	Intern
Scenario 1	≤0.01	≤0.05	≤0.15
Scenario 2	≤0.1	≤0.3	≤1

**Table 9:** Difference among role distance from CV and given margin.

	Roles	Subject A	Subject B
Scenario 1	Manager	0.01	0.05
	Employee	0.02	−0.02
	Intern	−0.04	−0.05
Scenario 2	Manager	−0.08	−0.04
	Employee	−0.23	−0.27
	Intern	−0.04	−0.9
